# Factors Associated with Knowledge, Attitudes, and Practices Related to Oral Care Among the Elderly in Hong Kong Community

**DOI:** 10.3390/ijerph17218088

**Published:** 2020-11-02

**Authors:** Florence M.F. Wong

**Affiliations:** School of Nursing, Tung Wah College, Hong Kong, China; florencewong@twc.edu.hk; Tel.: +852-3468-6838

**Keywords:** elderly, oral care, knowledge, attitudes, practices, community

## Abstract

Background: The oral health of elderly populations is a significant concern, as it is closely linked to general health and health-related quality of life. Poor oral health exacerbates oral diseases, leading to an increased risk of non-communicable diseases and self-care dependence, particularly in the elderly, worldwide. Knowledge, attitudes, and practices (KAP) play influential roles in individual oral care. However, the evidence of KAP related to oral care among the elderly is still inadequate. Objective: This study aimed to examine KAP and their associated factors related to oral care among the elderly community. Methods: A cross-sectional descriptive design was used. The eligible subjects were recruited, using convenience sampling and snowball sampling. Results: A total of 385 elderly individuals were included, and 51.4% were women. The mean age was 71.66 (SD 6.28). Knowledge was reportedly poor, as more than 50% elderly answered several questions incorrectly. The mean attitude and practices scores were 44.94 (SD 6.33) and 68.90 (SD 10.44), respectively. There was a significant correlation among knowledge, attitudes, and practices (*p* < 0.001). Multivariable regression analysis revealed numerous factors had remarkably significant association with knowledge [R^2^ = 0.30, F (4,380) = 11.96, *p* < 0.001], attitudes [R^2^ = 0.28, F (6,378) = 9.27, *p* < 0.001], and practices [R^2^ = 0.31, F (5,379) = 12.34, *p* < 0.001], particularly education levels, full-time employment, and self-care independence. Conclusions: Based on the KAP theoretical model, KAP are closely interrelated. Identified factors associated with KAP are useful to understand at-risk groups. Elderly individuals at lower education levels, with poor family support and inadequate self-care independence, have poor KAP related to oral care. Therefore, this study improves insight for health promotion developers, suggesting that more attention should be paid to at-risk elderly groups’ oral health. To enhance participation in dental care and improve oral care performance in the elderly, educational program containing oral health knowledge should be offered to at-risk groups. Family support and involvement are also important for improving oral care among elderly individuals.

## 1. Introduction

### 1.1. Background

Oral health has gained increasing attention worldwide, particularly regarding the elderly, as poor oral health causes non-communicable diseases that can, in turn, lead to increased mortality, lower health-related quality of life (HRQoL), and increased healthcare expenses [1]. With advances in healthcare technology and better services, many elderly people enjoy a prolonged life expectancy. However, the ageing population has garnered significant concern worldwide because the demographic is more susceptible to chronic illnesses leading to physical disability, psychosocial distress, and poor HRQoL [2,3]. As a result, greater attention has been paid to the rehabilitation and enhancement of self-care management [4]. Promotion for oral health among elderly individuals has thus been emphasized in recent decades. Oral health and dental care schemes have been developed by the governments of various countries, such as Japan and the UK [5]. Healthcare services for elderly people living in communities have been further developed, to improve their health and quality of life [6,7].

In general, elderly people, particularly aged 65 and above, are more likely to experience various degrees of oral and dental problems that adversely affect their physical and psychosocial well-being [8]. The most common oral and dental problems are gum bleeding, gingivitis, dental caries, oral infectious diseases, oral mucosal lesions, loss of natural teeth, and severe dental conditions [2]. Those conditions disrupt normal oral and dental function, such as chewing and speaking, leading to further physical and psychosocial problems, such as malnutrition, social isolation, and low self-esteem and low image [9,10]. At the age of retirement, elderly people face physical and socioeconomic changes that may affect their oral care and health. Evidence shows that many elderly people aged 65 and above have multiple or various degrees of oral diseases and related complications, such as missing natural teeth, dental caries, periodontitis, gum bleeding, infectious diseases, and cancer, particularly in those who are physically limited and more self-dependent [11]. In recent decades, oral health in elderly people has drawn significant attention. Their dental health is an important indicator of HRQoL and general health [10]. Thus, the Hong Kong government established an oral-health promotional-activity scheme to provide dental treatment with sponsorship. In 2019, the Community Care Fund Elderly Dental Assistance Programme was launched to provide a variety of dental services—including free removable dentures, dentures, crowns, bridges, scaling, fillings, root canal treatments, and tooth extraction—to eligible elderly people aged 65 or above [12]. Similar dental services can be found in other countries, such as Japan and UK [5]. However, enhanced oral health is subject to individual knowledge, attitudes, and practices (KAP) related to oral care. There is still an insufficient understanding of KAP related to oral care among elderly populations, particularly those who have received the Dental Assistance Fund supported by the government.

### 1.2. Literature Review

Most elderly individuals, particularly those who are self-care dependent and/or have a physical disability, are found to have poor oral health [13,14]. As aforementioned, KAP have been evidenced to influence an individual’s oral health [14,15]. The following section will thus break down reports of improper knowledge, attitudes, and practices, respectively. First, in both local and overseas studies, elderly people have shown inadequate knowledge of oral care [9,10,11,16,17]. Most elderly individuals do not use proper dental tools for their dental care, including dental floss and toothbrush [16,18]. Furthermore, many elderly people do not have knowledge about oral diseases and oral care. Only about 35% of elderly people report changing their toothbrush regularly [9,10,11]. 

Second, poor attitudes, based on personal perceptions on oral care and oral health, influence oral health among elderly people significantly. For instance, almost half of elderly people recognize that lost teeth can be replaced by denture, but they ignore the importance of oral care [11]. Fewer than 50% of elderly people report regular dental checks and proper oral care habit, such as tooth-brushing twice a day [16]. The high cost of dental consultations and the extended travel distances to dental clinics could be factors associated with poor oral and dental health among elderly people [19,20], who often only visit dentists when they discover oral or dental problems [20].

Finally, proper oral practices must be followed to maintain oral health. However, most elderly people use toothpicks instead of dental floss [21]. They also do not generally maintain the habit of brushing their teeth twice a day and rinsing their mouth each time [16]. As a result, serious oral problems are present, such as deep gum pocket, periodontitis, and dental erosion [10].

Based on the knowledge–attitudes–practices model developed by Zeng et al. [18], improved knowledge increases an individual’s awareness of a specific construct. Positive attitudes can be attributed to adequate levels of knowledge, leading to improved motivation. Practices are related to self-care based on levels of knowledge and attitudes. Since KAP are interrelated, an investigation of poor oral health in the elderly population was necessary. Understanding this demographic’s KAP makes it possible to develop strategies to improve oral health among elderly people. Moreover, factors associated with KAP related to oral care play influential roles in oral health. Past studies and reviews reported various factors influencing oral health among elderly people, including age, gender, number of medications [22,23,24], and self-care dependence [21,25]. Due to inadequate evidence to support most current findings, further study on identifying factors has been encouraged. Therefore, this study aimed to examine the KAP related to oral care and to identify factors associated with the KAP related to oral care among elderly people. Hopefully, the results of this study can help develop promotional guideline or protocols for managing the oral health of this at-risk vulnerable elderly population.

### 1.3. Aims

This study’s aims were (1) to examine the KAP related to oral care and (2) to identify factors associated with the KAP related to oral care among elderly people.

## 2. Materials and Methods 

### 2.1. Design

A descriptive cross-sectional design was conducted. 

### 2.2. Study Sample

Eligible participants were recruited through convenience sampling and snowball sampling. Inclusion criteria comprised elderly people (1) aged 65 or older, (2) who were Hong Kong residents, and (3) who were able to communicate in Chinese and English. Elderly people who had cognitive problems or were dependent for oral care were excluded. This study’s sample size calculation was performed by using the Survey System of Creative Research Systems. The estimated total population of individuals aged 65 and older, as reported by the Census and Statistics Department in 2019 [26], was 1,324,600. A total of 384 elderly participants were able to reach a 95% confidence level and a 5% margin of error.

### 2.3. Data Collection

An approval (NUR/SRC/20200106/034) was sought from the research ethics committee of the study educational institution prior to commencement of the study. Implied consent was taken when participants agreed to complete the questionnaires. The questionnaires consisted of a demographic form and questions pertaining to KAP related to oral care among elderly people. Data were collected via face-to-face interviews, telephone interviews, or online Google questionnaire, from 1 January to 30 June 2020. Data collection was adapted to an online Google questionnaire with verbal recordings of each item due to the COVID-19 pandemic. The completion of questionnaires took about 20 min.

### 2.4. Instrument, Knowledge, Attitudes, and Practices of Oral Care among Elderly People

To understand more about KAP with regards to oral care among elderly people, a questionnaire was developed based on previous similar studies [27] and comments from experts in dentistry (two dental hygienists, two dentists, and one university professor in faculty of dentistry). A content validation was conducted. The items were revised until the content validity index of the final version was 0.95, indicating excellent reliability. The questionnaire included knowledge, attitudes, and practices related to oral care among elderly people (Supplementary Materials Table S1). The questionnaire consists of three parts, each dedicated to one component of KAP. There was a total of 15 items for knowledge, using ‘true’, ‘false’, and ‘don’t know’ choices. The score range was 0 to 30. There were 12 items regarding attitudes and 20 items for practices related to oral care among elderly people, all employing the 5-Likert scale ranging from strongly disagree (score 0) to strongly agree (score 5). The score ranges of the attitudes and practices were 0–60 and 0–100, respectively. A pilot study was conducted with 20 participants to determine the availability of participants, as well as flexibility and practicality of the study. The internal validity with test-retest reliability was determined. The Cronbach’s alphas of the overall scores of the pilot study were 0.87, demonstrating very good reliability. 

### 2.5. Statistical Analysis

The SPSS v25.0 (IBM Corporation, Armonk, New York, USA) was used for data analysis. The normality of the continuous variables was assessed by their skewness statistics and normal Q–Q plots. There was no continuous variable found to violate the normality assumption. Descriptive statistics were applied to summarize and present the explanatory variables related to participants’ characteristics and the outcome variables (KAP). Associations between the outcome variables (KAP) and demographic characteristics (age, gender, educational level, marital status, employment status, and others) were assessed by Chi-square or univariate analyses using Pearson’s correlation coefficient, independent-samples t-test, or one-way ANOVA (depending on the level of measurement of the outcome variables) in identifying variables for multivariable regression. The variables with *p*-values < 0.25 were entered in the model of stepwise multivariable regression analysis to delineate factors independently associated with each outcome of KAP. The presence of collinearity was assessed by tolerance. Model adequacy will be assessed by examining the scatter plot of standardized residuals against the predicted values, and the normal probability plot of residuals. All statistical tests are two-sided, and a *p*-value < 0.05 is considered statistically significant.

### 2.6. Ethical Considerations

Approval was sought from the research ethics committee of the study educational institute. Participants were requested to sign an informed consent form once they had agreed to participate in the study. They were assured that all data related to their personal information would be kept strictly confidential and anonymous.

## 3. Results

### 3.1. Demographic Characteristics (Table 1)

A total of 385 Hong Kong Chinese elderly individuals aged from 65 to 92 were recruited based on the selection criteria. The mean age was 71.66 (SD 6.28) years old. Of the 385 elderly individuals, 48.6% (n = 187) were men, 61.0% (n = 235) were married, 57.6% (n = 194) had received secondary or higher educational level, 86.5% (n = 333) were retired or unemployed, 13.5% (n = 52) had an occupation, 83.9% (n = 323) were fully self-care independent, and 35.4% (n = 140) had a long-term illness. Table 1 shows these demographic characteristics.

### 3.2. Knowledge, Attitudes, and Practices (Table 2)

Table 2 shows the results of KAP of elderly people in oral care. The means of KAP were 8.7 (SD 2.25) (range from 0 to 30), 44.94 (SD6.3) (range from 0 to 60), and 68.90 (SD10.44) (range from 0 to 100), respectively. The mean of overall KAP was 122.54 (SD15.52).

Knowledge was relatively poorer than attitudes and practices related to oral care. The elderly participants were likely to harbor several misconceptions specifically. For instance, participants revealed that they were misinformed about the following statements: ‘As people get old, they naturally have loose teeth’ (incorrect 86.5%), ‘It’s normal for elderly people to have pain and sores in their mouth’ (incorrect 66.5%), ‘If dentures don’t fit well, they can cause oral cancer’ (incorrect 64.7%), ‘Older people with dry mouths get more cavities’ (incorrect 62.9%), and ‘The most common cause of dry mouth is medications’ (incorrect 51.4%).

### 3.3. Associations among Knowledge, Attitudes, and Practices of Elderly People in Oral Care (Table 3)

There were significant correlations found among KAP (*p* < 0.001). The results of the associations among KAP are shown in Table 3.

### 3.4. Factors Associated with Knowledge, Attitudes, and Practices (Table 4)

The results of multivariate regression for factors associated with the KAP of elderly people in oral care are summarized in Table 4. The multivariate regressions identified factors that were significantly associated with KAP related to oral care among elderly people. The results showed that receiving higher educational levels [primary education (B = 1.32, SE = 0.23, *p* < 0.001) and secondary education (B = 1.26, SE = 0.44, *p* = 0.005), tertiary education (B = 2.75, SE = 1.24, *p* = 0.027)], and being fully independent (B = 0.64, SE = 0.30, *p* = 0.034) had significant positive correlations with knowledge of oral care [R^2^ = 0.30, F (4,380) = 11.96, *p* < 0.001].

The results also showed that being of the age 71–75 (B = 2.52, SE = 0.83, *p* = 0.002), being married (B = 2.50, SE = 0.68, *p* < 0.001), being single (B = 3.16, SE = 1.20, *p* = 0.009), being employed (B = 2.55, SE = 1.12, *p* = 0.023), and being fully self-care independent (B = 2.01, SE = 0.84, *p* = 0.017) had a significant positive correlations, but lower education [primary education or below (B = −2.31, SE = 0.65, *p* < 0.001)] had significant negative correlation with attitudes of oral care [R2 = 0.28, F (6,378) = 9.27, *p* < 0.001].

Receiving higher educational levels [primary education (B = 5.95, SE = 1.05, *p* < 0.001) and secondary education (B = 6.43, SE = 2.01, *p* = 0.002), tertiary education (B = 17.95, SE = 5.68, *p* = 0.002)], and being employed (B = 4.16, SE = 1.80, *p* = 0.021) had positive correlations, but being separated had a significant negative correlation with practices of oral care [R^2^ = 0.31, F (5,379) = 12.34, *p* < 0.001].

### 3.5. Factors Associated with Knowledge, Attitudes, and Practices Using Univariate Analysis (Table 5)

To understand more about the effects of each parameter on the measure outcomes (KAP) after age and gender were adjusted with 0.9% confidence interval, univariate regression analysis was conducted. The results are illustrated in Table 5.

## 4. Discussion

This study aimed to examine the KAP related to oral care, as well as the factors associated with oral care, among an elderly population. The results have expanded the current understanding of the KAP related to oral care among elderly people and their associated factors. According to the KAP theoretical model, there are significant and close associations among KAP in the present study. It is important to note that an enhancement in knowledge of oral care has a significant positive effect on the attitudes towards and practices of oral care in elderly populations [16,28].

The present study results showed that attitudes towards and practices of oral care were generally more developed than knowledge of oral care. However, knowledge was poor due to unclear perception of oral care among elderly people. Such a finding is comparable to a similar study [18] conducted in elderly populations. The researchers explained that the elderly participants had basic knowledge of oral care. However, they did not have knowledge of oral problems such as periodontal diseases. Similarly, in this present study, most of the elderly people perceived that oral problems, such as pain and loose teeth, in elderly people is natural; this can be an ingrained belief. Most of the Chinese elderly people believed that they could use dentures to replace tooth loss [11,16]. In terms of denture care, the Chinese elderly people may not be aware of severe potential oral health consequences due to unfit dentures, such as oral cancer [29,30]. Due to their misunderstanding or overconfidence in ingrained misconceptions, elderly people may not be able to maintain their oral health properly. Most of the Chinese elderly participants in this study did not have long-term illness. Thus, they may not be aware of oral problems attributed to dry mouth and/or medications. Many medications have the side effect of dry mouth, which leads to further oral problems [22,23,24]. Maintaining better oral health with adequate moisture in the mouth, such as regular drinking water or rinsing the mouth, is fundamental knowledge [16,18]. However, such basic knowledge for oral health, including frequency of tooth-brushing and regular dental visits, worsens among old age groups [31,32]. In the Chinese population, elderly people were found to have poor dental health habits and attitudes, including the lack of using dental floss or attending regular dental checkups [33]. They often only visit dental consultation when they have an existing oral problem, such as oral pain or gum bleeding [11]. Appropriate oral care knowledge must be delivered to elderly people for the maintenance of oral health and the prevention of all oral problems, which can affect eating problems, weight loss and nutritional problems, speech difficulty, and psychosocial distress [34,35]. Therefore, understanding this poor knowledge among elderly people helps increase awareness for healthcare promotion developers’ strategies, such as joining in oral care programs, to provide appropriate knowledge of oral care to all elderly people [36]. In the Chinese culture, it is especially important to approach the elderly people and invite them to join the educational oral care programs with great respect [36,37].

The studied factors can help identify at-risk populations for poor oral care. Higher education was found to have a significant positive association with knowledge and practices, but lower education had a negative association with attitudes of oral care. Undoubtedly, elderly people who have received higher education are more likely to search relevant information about oral problems and preventive measures, such as using dental floss and changing their toothbrush regularly, via various channels. Based on the KAP theoretical model, better knowledge of oral health increases positive attitudes and behaviors [18,38]. When elderly people are able to receive adequate opportunities to enhance their knowledge, their motivation, and responsibility to maintain their oral health increases [18]. It can be explained that poor knowledge can negatively affect attitudes and practices, leading to poor motivation and performance for oral health.

Elderly people who are fully self-care independent are more likely to garner more knowledge and have better attitudes towards oral care and oral health. They are able to be more aware of the importance of oral health and be more responsible for their own oral health. Elderly people who are more self-care independent are more active to participate in activities that promote oral health [39,40]. They are more willing to learn new and appropriate knowledge of oral care/health and gain information about oral-care services. Consequently, their attitudes towards oral care improve accordingly [15].

Being employed was found to have positive association with attitudes and practices related to oral care. Employment is one of the important social networks. Maintaining oral health is important for social relationships. Elderly people who are being employed have financial support. Having an income is positively correlated with oral care because elderly people are able to choose oral care services and take measures to improve their oral health [41]. This implies that elderly people who are in lower socioeconomic classes have difficulty in affording dental services, especially if they have no access to health insurance coverage for dental care. A lower income and loss of insurance for dental coverage, particularly after retirement, are the main reasons that elderly people are unwilling to attend dental visits [42,43]. It is important that elderly people receive knowledge of and participate in oral-health-related activities during dental care visits [44]. In this sense, fewer dental-care visits accelerate oral problems in elderly people. Therefore, dental care supported or subsidized by the government or other sponsorship organizations can help improve overall oral health in elderly population.

Individuals between the ages of 71 and 75 were found to have a significant positive association with attitudes of oral care among elderly people. Since most elderly people start their retirement at age 65, at the initial retirement period, elderly people are more likely to have more psychosocial adaptation as their roles and social network change, especially with their colleagues. Financially, elderly people may also need to adapt to changed economic status after retirement. Therefore, better attitudes of oral care in elderly people aged 71 to 75 may be due to their adjustment after retirement. Although age was not found as a factor associated with practices of oral care, another study found that increased age was, in fact, correlated with significantly lower oral health practices [31].

Moreover, elderly people who are married or single have better attitudes of oral care, which may be due to the fact that they have received support from their family or friends. However, elderly people who have experienced divorce or separation have poorer practices of oral care. This may be due to inadequate social support, particularly from family. Family involvement has a positive effect on oral health and utilization of dental services [45]. This implies, in turn, that unfortunate relationships negatively impact oral care. Separated relationships reduce social support, which lowers self-awareness of oral health [46]. Family completeness is crucial in the Chinese context, because one values family and emphasizes mutual support among family members [47].

Better oral health is subject to effective oral care with proper tooth-brushing and oral cleaning [21,48] and regular yearly dental checkups [42,43]. Primary care for oral health is crucial for the elderly population, to increase their awareness of oral care and oral health [38]. Educational programs should be provided to elderly people and their family to improve oral care practices through various channels, such as TV, mass media, posters, and pamphlets. It is important to note that these educational programs should consider the at-risk elderly groups for effective implementation of oral care by controlling and reducing the vulnerable effects arising from negative factors, but also enhancing the effects of positive factors to further improve KAP related to oral care contributing to oral health. More importantly, knowledge exchange and understanding of perspectives of oral care among elderly people and their families can help develop suitable educational content [49]. Annual dental checkups facilitate oral health and provides immediate dental-related treatment to the elderly population. As the dental promotional healthcare scheme for elderly people is subsidized by the government and dental clinics cooperates with the government to provide dental services to elderly people, oral health will likely improve. The locations of those clinics must be easily accessible to elderly people.

###  Strengths and Weaknesses

This study, since it used a cross-sectional design, may not provide causal inferences between KAP and the associated factors. Therefore, longitudinal studies are recommended to detect changes across study periods. The KAP model used in the present study may not be able to understand more about the behavioral changes. Other models, such as COM-B by West and Michie [50], can be used in future studies. A case study or qualitative study may help better understand perspectives of performing oral care and how family support is associated with oral care among elderly people. Better attitudes to oral care was found in those aged 71 to 75. Further study may be needed to understand more about this finding. However, this present study’s results may not be generalizable to other age groups and non-Chinese societies, due to cultural disparities and differences in healthcare service policies, as only an elderly Chinese population aged 65 or older were recruited. This survey was used to collect subjective results about KAP related to oral care among elderly people, but oral examination performed by a dental expert can provide more valuable insight into oral care. Some factors with insignificant results may be due to the small sample size of the specific groups, such as having long-term illnesses and taking long-term medications. To understand more about their effects on the KAP related to oral care, a larger sample of this specific group should be examined in future studies. Moreover, this study did not include elderly people in long-term-care institutions. Oral problems in institutionalized residents may be a concern because of their limited physical and self-care abilities. The oral care of elderly people performed by institutionalized caregivers may need to be surveyed. Due to the COVID-19 pandemic, an online questionnaire was adopted to replace face-to-face interviews. It may be difficult for elderly people who have vision problems or who are unfamiliar with using the internet or digital devices. An audio-recording of each question or a larger font size would help elderly people understand the questions more easily. Data collection using snowball sampling not only reduces the control by the researchers but also increases sampling bias, as the subjects could share the same characteristics.

## 5. Conclusions

The value of the present study is to provide new information about the KAP related to oral care among elderly people among the surveyed target group (aged 65 or older) and identify associated positive and negative factors influencing the KAP related to their oral care. Identified factors were useful to identify at-risk groups and to improve strategies of promoting oral care in elderly people. Negative factors can serve as barriers to the maintenance of oral health. Although the oral care scheme for elderly people has been subsidized by the government for years, the self-oral care can indirectly be evaluated based on the current study. Elderly people after retirement needs more family support and self-care ability for their oral health. Healthcare professionals should involve family to encourage their at-risk family elderly members to participate in the dental-care promotional program. Both government and healthcare policymakers need to coordinate efforts to improve awareness and participation in education about oral health to eligible population through various promotional channels. The government must continue the subsidized support of oral care among elderly people and the monitoring of the oral health of this specific population, particularly those at a lower socioeconomic level.

## Figures and Tables

**Table 1:** Demographic characteristics (n = 385).

Demographics	n (%)/Mean (SD)
**Gender**	
Male	187 (48.6%)
Female	198 (51.4%)
Age *	71.66 (6.28)
65–70	228 (59.2%)
71–75	65 (16.9%)
76–80	45 (11.7%)
81–85	32 (8.3&)
86–90	12 (3.1%)
>90	3 (8.0%)
**Marital Status**	
Single	32 (8.3%)
Married	235 (61.0%)
Separated	18 (4.7%)
Divorced	33 (8.6%)
Widowed	67 (17.4%)
**Educational Level**	
Illiterate	67 (17.4%)
Primary	124 (32.2%)
Secondary	164 (42.6%)
Tertiary	27 (7.0%)
Above tertiary	3 (8.0%)
**Employment Status**	
Unemployed	9 (2.3%)
Employed	33 (8.6%)
Self-employed	19 (4.9%)
Retired	267 (69.4%)
Housewife/Househusband	57 (14.8%)
**Self-Care Independence**	
Fully independence	323 (83.9%)
Partial independence	62 (16.1%)
Long-Term Illness	
No	245 (63.6%)
Yes	140 (35.4%)
**Number of Medications Taken**	
None	246 (63.9%)
1–3	79 (20.5%)
4–6	44 (11.4%)
7–9	9 (2.3%)
≥10	7 (1.8%)

Data marked with * are presented as mean (SD), whereas the others are presented as frequency (%).

**Table 2:** Knowledge, attitudes, and practices (KAP) of elderly people related to oral care (n = 385).

	Mean	SD
Knowledge (0–30)	8.70	2.25
Attitudes (0–60)	44.94	6.33
Practices (0–100)	68.90	10.44
Overall KAP (0–190)	122.54	15.52

**Table 3:** Correlations among KAP of elderly people in oral care (n = 385).

	Correlation Coefficient			Significance (*p*)
	Knowledge (K)	Attitudes (A)	Practices (P)	
**Knowledge**	-	0.40	0.47	<0.001
**Attitudes**	0.40	-	0.40	<0.001
**Practices**	0.47	0.40	-	<0.001

**Table 4:** Results of multivariate regression for factors associated with KAP related to oral care among elderly people (n = 385).

	Knowledge			Attitudes			Practices		
	B	SE	*p*	B	SE	*p*	B	SE	*p*
Aged 71–75				2.52	0.83	0.002 *			
Married				2.50	0.68	<0.001 **			
Single				3.16	1.20	0.009 *			
Separated							−6.34	2.36	0.007 *
Primary education				−2.31	0.65	<0.001 **			
Secondary education	1.32	0.23	<0.001 **				5.95	1.05	<0.001 **
Tertiary education	1.26	0.44	0.005 *				6.43	2.01	0.002 *
Above tertiary education	2.75	1.24	0.027 *				17.95	5.68	0.002 *
Employed				2.55	1.12	0.023 *	4.16	1.80	0.021 *
Fully independence	0.64	0.30	0.034 *	2.01	0.84	0.017 *			

B, the regression coefficient; SE, standard error of the regression coefficient; *p*, significance; ** *p* < 0.005; * *p* < 0.05.

**Table 5:** Results of univariate regression for each factor associated with KAP of oral care among elderly people (n = 385).

	Knowledge				Attitudes				Practices			
	B	SE	*p*	*95%CI*	B	SE	*p*	*95%CI*	B	SE	*p*	*95%CI*
**Gender**												
Female	0.17	0.22	0.438	−0.27 to 0.61	0.54	0.65	0.404	−0.73 to 1.82	1.39	1.08	0.199	−0.73 to 3.52
Male (reference group)	0											
**Age**												
65–70	−0.87	1.26	0.492	−3.44 to 1.61	1.45	3.50	0.678	−5.44 to 8.34	−4.97	5.84	0.395	−16.46 to 6.51
71–75	−0.82	1.28	0.523	−3.32 to 1.69	3.69	3.55	0.300	−3.29 to 10.66	−5.16	5.91	0.384	−16.78 to 6.47
76–80	−0.62	1.28	0.628	−3.14 to 1.90	2.15	3.56	0.546	−4.85 to 9.15	−6.20	5.94	0.297	−17.88 to 5.48
81–85	−1.27	1.30	0.326	−3.82 to 1.27	1.35	3.59	0.708	−5.71 to 8.41	−6.25	5.99	0.297	−18.02 to 5.52
86–90	−2.00	1.38	0.148	−4.72 to 0.71	−1.43	3.83	0.709	−8.96 to 6.10	−7.09	6.38	0.267	−19.64 to 5.46
>90 (reference group)	0				0				0			
**Marital Status**												
Single					2.47	1.32	0.062	−0.13 to 5.06	0.001	2.20	1.00	−4.23 to 0.00
Married					1.78	0.87	0.043	0.06 to 3.49	0.10	1.46	0.494	−1.86 to 3.86
Separated					−1.97	1.61	0.222	−5.14 to 1.20	−5.70	2.69	0.035	−10.99 to −0.42
Divorced					0.76	1.30	0.557	−1.79 to 3.32	0.62	2.17	0.775	−3.64 to 4.88
Widow (reference group)					0				0			
**Educational Levels**												
Illiterate (reference group)	0				0				0			
Primary education	0.30	0.34	0.371	−0.36 to 0.96	1.32	0.94	0.159	−0.52 to 3.16	0.71	1.56	0.650	−2.36 to 3.77
Secondary education	−1.09	0.35	0.002	−1.78 to −0.41	−1.59	0.99	0.108	−3.52 to 0.35	−4.92	1.63	0.003	−8.13 to −1.71
Tertiary education	−1.09	0.52	0.037	−4.97 to 0.06	−3.43	1.47	0.020	−6.32 to 0.55	−5.46	2.43	0.026	−10.24 to −0.67
Above tertiary education	−2.46	1.28	0.055	−4.96 to 0.05	−5.28	3.54	0.137	−12.25 to 1.69	−16.66	5.91	0.005	−28.27 to −5.05
**Employment Status**												
Unemployed (reference group)					0				0			
Employed					−6.64	2.29	0.004	−11.14 to −2.13	−1.57	3.82	0.681	−9.08 to 5.94
Self-employed					−2.94	2.42	0.224	−7.69 to 1.81	0.64	4.03	0.874	−7.29 to 8.56
Retired					−4.35	2.07	0.036	−8.42 to −0.28	2.57	3.45	0.456	−4.21 to 0.35
Housewife/househusband					−5.60	2.21	0.012	−9.95 to −1.26	2.56	3.68	0.488	−4.68 to 9.80
**Self-Care Dependence**												
Fully dependence	−0.65	0.31	0.036	−0.25 to −0.04	1.74	0.65	0.404	−0.73 to 1.82				
Partial dependence (reference group)	0				0							
	Adj. R^2^ = 0.102; F-statistics of the corrected model (F = 4.98, df = 11; *p* < 0.001)				Adj. R^2^ = 0.132; F-statistics of the corrected model (F = 4.08, df = 19; *p* < 0.001)				Adj. R^2^ = 0.113; F-statistics of the corrected model (F = 3.73, df = 18; *p* < 0.001)			

Parameters adjusted by age and gender with 95% confidence interval; B, the regression coefficient; SE, standard error of the regression coefficient; *p*, significance; CI, confidence interval; df, degree of freedom.

## Supplementary Materials

The following are available online at https://www.mdpi.com/1660-4601/17/21/8088/s1. Table S1: The knowledge, attitudes, and practices questionnaire. Table S2: The STROBE statement—checklist of items for cross-sectional studies.
