# Supplementary material for: Factors Associated with Knowledge, Attitudes, and Practices Related to Oral Care Among the Elderly in Hong Kong Community
Source: Int J Environ Res Public Health. 2020 Nov 2;17(21):8088. doi: 10.3390/ijerph17218088 (PMC7672548; doi:10.3390/ijerph17218088)
Supplement: Supplementary file 1 [file ijerph-17-08088-s001.zip › ijerph-927767 - Supp. materials/KAP questionnaire.doc]

**Table 1. Knowledge, attitude, and practice of oral care among elderly in community Knowledge (please tick the appropriate box)**

|  | **No** |  |  | **Items** | | | | | | | | | | **Yes** | | | | **No** | **Don’t know** | | | |
| --- | --- | --- | --- | --- | --- | --- | --- | --- | --- | --- | --- | --- | --- | --- | --- | --- | --- | --- | --- | --- | --- | --- |
|  |  |  |  |  |  |  |  |  |  |  |  |  |  |  |  |  |  |  |  |  |  |  |
| **1** | |  |  | **Oral health is directly related to general health.** | | | | | | | | | |  | | | |  |  | | | |
|  |  |  |  |  |  |  |  |  |  |  |  |  |  |  |  |  |  |  |  |  |  |  |
| **2** | |  |  | **You can chew just as well with denture tooth as with your natural teeth.** | | | | | | | | | |  | | | |  |  | | | |
|  |  |  |  |  |  |  |  |  |  |  |  |  |  |  |  |  |  |  |  |  |  |  |
| **3** | |  |  | **When gums bleed during brushing, it is better leave them alone.** | | | | | | | | | |  | | | |  |  | | | |
|  |  |  |  |  |  |  |  |  |  |  |  |  |  |  |  |  |  |  |  |  |  |  |
| **4** | |  |  | **Older people with dry mouth get more cavities.** | | | | | | | | | |  | | | |  |  | | | |
|  |  |  |  |  |  |  |  |  |  |  |  |  |  |  |  |  |  |  |  |  |  |  |
| **5** | |  |  | **The most common cause of dry mouth is medication.** | | | | | | | | | |  | | | |  |  | | | |
|  |  |  |  |  |  |  |  |  |  |  |  |  |  |  |  |  |  |  |  |  |  |  |
| **6** | |  |  | **Older people with teeth need to use fluorides.** | | | | | | | | | |  | | | |  |  | | | |
|  |  |  |  |  |  |  |  |  |  |  |  |  |  |  |  |  |  |  |  |  |  |  |
| **7** | |  |  | **Mouth rinsing is a good alternative to daily tooth brushing.** | | | | | | | | | |  | | | |  |  | | | |
|  |  |  |  |  |  |  |  |  |  |  |  |  |  |  |  |  |  |  |  |  |  |  |
| **8** | |  |  | **Older people with no teeth need to have regular dental consultation.** | | | | | | | | | |  | | | |  |  | | | |
|  |  |  |  |  |  |  |  |  |  |  |  |  |  |  |  |  |  |  |  |  |  |  |
| **9** | |  |  | **Dentures should be removed for few hours every day.** | | | | | | | | | |  | | | |  |  | | | |
|  |  |  |  |  |  |  |  |  |  |  |  |  |  |  |  |  |  |  |  |  |  |  |
| **10** | |  |  | **Dentures those do not fit well can cause oral cancer.** | | | | | | | | | |  | | | |  |  | | | |
|  |  |  |  |  |  |  |  |  |  |  |  |  |  |  |  |  |  |  |  |  |  |  |
| **11** | |  |  | **It is normal for elderly people to have pain and sores in their mouth.** | | | | | | | | | |  | | | |  |  | | | |
|  |  |  |  |  |  |  |  |  |  |  |  |  |  |  |  |  |  |  |  |  |  |  |
| **12** | |  |  | **Older people with cognitive problem should leave them alone for daily mouth**  **care.** | | | | | | | | | |  | | | |  |  | | | |
|  |  |  |  |  |  |  |  |  |  |  |  |  |  |  |  |  |  |  |  |  |  |  |
|  |  |  |  |  |  |  |  |  |  |  |  |  |  |  |  |  |  |  |  |  |  |  |
| **13** | |  |  | **Dental checkups are as important as medical checkups.** | | | | | | | | | |  | | | |  |  | | | |
|  |  |  |  |  |  |  |  |  |  |  |  |  |  |  |  |  |  |  |  |  |  |  |
| **14** | |  | **Older people can have loose teeth if they remain dirty.** | | | | | | | | | | |  | | | |  |  | | | |
|  |  |  |  |  |  |  |  |  |  |  |  |  |  |  |  |  |  |  |  |  |  |  |
| **15** | |  | **As people get old, they naturally have loose teeth.** | | | | | | | | | | |  | | | |  |  | | | |
|  |  |  |  |  |  |  |  |  |  |  |  |  |  |  |  |  |  |  |  |  |  |  |
|  | **Attitude (please tick the appropriate box)** | | | | | | | | | | | | | | | | | | | | | |
|  |  |  |  |  |  |  |  |  |  |  |  |  |  |  |  |  |  |  |  |  |  |  |
|  |  |  |  |  |  |  |  |  |  |  |  |  |  |  |  |  |  |  |  |  |  |  |
|  |  |  |  |  |  |  |  |  |  |  |  |  |  |  |  |  |  |  |  |  |  |  |
|  |  |  |  | **Items** | |  |  | |  |  |  |  |  |  |  |  |  | |  |  |  | |
|  | **No** |  |  |  |  |  | **Strongly** |  |  | **Somewhat** |  |  | **Neural** | |  |  | **Somewhat** | |  |  | **Strongly** |  |
|  |  |  |  |  |  |  | **agree** |  |  | **agree** |  |  |  |  |  |  | **disagree** | |  |  | **disagree** |  |
|  |  |  |  |  |  |  |  |  |  |  |  |  |  |  |  |  |  |  |  |  |  |  |
|  |  |  |  |  |  |  |  |  |  |  |  |  |  |  |  |  |  |  |  |  |  |  |
|  | **1** | | **Elderly people who have dentures need oral** | | |  | | |  | | |  | | | |  | | | |  | | |
|  |  |  | **care.** | | |  |  |  |  |  |  |  |  |  |  |  |  |  |  |  |  |  |
|  |  | |  | | |  |  |  |  |  |  |  |  |  |  |  |  |  |  |  |  |  |
|  | **2** | | **I want to enhance my knowledge on oral care.** | | |  | | |  | | |  |  | | |  | | | |  | | |
|  |  | |  | | |  |  |  |  |  |  |  |  |  |  |  |  |  |  |  |  |  |
|  | **3** | | **Oral care training is good for me to perform** | | |  | | |  | | |  | | | |  | | | |  | | |
|  |  |  | **better oral care.** | | |  |  |  |  |  |  |  |  |  |  |  |  |  |  |  |  |  |
|  |  | |  | | |  |  |  |  |  |  |  |  |  |  |  |  |  |  |  |  |  |
|  | **4** | | **I am able to assess my oral condition myself.** | | |  | | |  | | |  | | | |  | | | |  | | |
|  |  | |  | | |  |  |  |  |  |  |  |  |  |  |  |  |  |  |  |  |  |
|  | **5** | | **I brush my teeth twice a day.** | | |  | | |  | | |  | | | |  | | | |  | | |
|  |  | |  | | |  |  |  |  |  |  |  |  |  |  |  |  |  |  |  |  |  |
|  | **6** | | **Oral care is one of priority procedures for my** | | |  | | |  | | |  | | | |  | | | |  | | |
|  |  |  |  |  |  |  |  |  |  |  |  |  |  |  |  |  |  |  |  |  |  |  |

**health.**

7 **Oral care is an important part of my personal care.**

**8*	Oral care is an unpleasant task to me.**

9 **Maintaining a good oral hygiene can prevent oral infection and diseases.**

| **10** | | |  |  | | **I am willing to spend time on oral care.** | |  | |  | |  | |  | |  | |  | |  |  |  |  |  |  |  |  |  |
| --- | --- | --- | --- | --- | --- | --- | --- | --- | --- | --- | --- | --- | --- | --- | --- | --- | --- | --- | --- | --- | --- | --- | --- | --- | --- | --- | --- | --- |
|  |  |  |  |  | |  |  | |  | |  | |  | |  | |  | |  |  |  |  |  |  |  |  |  |  |
| **11** | | |  |  | | **I prefer doing oral care procedure.** | |  | |  |  |  |  |  |  |  |  |  |  | |  | |  | |  | |  | |
|  |  |  |  |  | |  |  | |  |  |  |  |  |  |  |  |  |  |  |  |  |  |  |  |  |  |  |  |
| **12** | | |  |  | | **I think oral care is my job, not the dentist's** | |  | |  | |  | |  | |  | |  | |  |  |  |  |  |  |  |  |  |
|  |  |  |  |  | | **job.** | |  |  |  |  |  |  |  |  |  |  |  | |  |  |  |  |  |  |  |  |  |
|  |  |  |  | |  | |  | |  |  |  |  |  |  |  |  |  |  |  |  |  |  |  |  |  |  |  |  |
|  | **Practice (please tick the appropriate box)** | | | | | | | | | | | | | | | | |  | |  |  |  |  |  |  |  |  |  |
|  |  |  |  |  |  |  |  |  |  |  |  |  |  |  |  |  |  |  | |  |  |  |  |  |  |  |  |  |
|  |  |  |  |  |  |  |  |  |  |  |  |  |  |  |  |  |  |  | |  |  |  |  |  |  |  |  |  |
|  |  |  | |  | |  |  | |  |  | |  | |  | |  | |  | |  |  |  |  |  |  |  |  |  |
|  |  | **No** |  |  | | **Items** |  | | **Strongly** | **Somewhat** | | **Neural** | | **Somewhat** | | **Strongly** | |  | |  |  |  |  |  |  |  |  |  |
|  |  |  |  |  | |  |  | | **agree** | **agree** | |  | | **disagree** | | **disagree** | |  | |  |  |  |  |  |  |  |  |  |
|  |  |  |  |  | |  |  | |  |  | |  | |  | |  | |  | |  |  |  |  |  |  |  |  |  |
| **1** | | |  |  | | **I will use the following *materials* to care my** | |  | |  | |  | |  | |  | |  | |  |  |  |  |  |  |  |  |  |
|  |  |  |  |  | |  |  |  | | | | | | | | | |  | |  |  |  |  |  |  |  |  |  |
|  |  |  |  |  | | **mouth:** | |  |  |  |  |  |  |  |  |  |  |  | |  |  |  |  |  |  |  |  |  |
|  |  |  |  |  | |  |  | |  |  |  |  |  |  |  |  |  |  |  |  |  |  |  |  |  |  |  |  |

1. 1 Manual toothbrush

1.2 Swabstick

1.3 Foam swabs

1.4 Mouth rinses

1.5 Moisturize of lips

**2**	**I will do the followings to maintain my oral**

**health.**

2.1 Brushing teeth

2.2 Brushing gums

2.3 Brushing tongue

2.4 Cleansing dentures

2.5 Performing mouthwash

**3**	**I will use the following *cleansing agents* to**

**clean my mouth.**

3.1 Thymol gargle

3.2 0.2% Chlorhexidine

3.3 Normal Saline

3.4 Sterile water

3.5 Boiled water

4 **I perform oral care every day and night.**
5 **I always take 10 minutes to perform oral care each time.**
6 **I always perform oral assessment before oral care.**

**7***	**When I am busy, I will skip my oral care.**

**8***	**I will not perform oral care if there is any**

**unpleasant feeling during the procedure.**

***reverse questions (will not show in the questionnaire for data collection)**
